# Regulation of NO-Generating System Activity in Cucumber Root Response to Cold

**DOI:** 10.3390/ijms26041599

**Published:** 2025-02-13

**Authors:** Małgorzata Reda, Katarzyna Kabała, Jan Stanisławski, Kacper Szczepski, Małgorzata Janicka

**Affiliations:** Department of Plant Molecular Physiology, Faculty of Biological Sciences, University of Wrocław, Kanonia 6/8, 50-328 Wrocław, Poland; malgorzata.reda@uwr.edu.pl (M.R.); katarzyna.kabala@uwr.edu.pl (K.K.); janski9999@gmail.com (J.S.); kacper.szczepski@kaust.edu.sa (K.S.)

**Keywords:** nitric oxide, low-temperature stress, nitrate reductase, nitric oxide synthase-like activity, ARC protein

## Abstract

Nitric oxide (NO) functions as a signaling molecule in plant adaptation to changing environmental conditions. NO levels were found to increase in plants in response to low temperatures (LTs). However, knowledge of the pathways involved in enhanced NO production under cold stress is still limited. For this reason, we aimed to determine the role of different NO sources in NO generation in cucumber roots exposed to 10 °C for short (1 d) and long (6 d) periods. The short-term treatment of seedlings with LT markedly increased plasma membrane-bound nitrate reductase (PM-NR) activity and induced the expression of three genes encoding NR in cucumber (*CsNR1-3*). On the other hand, long-term exposure was related to both increased cytoplasmic NR (cNR) activity and induced expression of the *CsARC* gene, encoding the amidoxime-reducing component (ARC) protein. The decrease in nitrite reductase (NiR) activity and the higher NO_2_^−^/NO_3_^−^ ratio in the roots of plants exposed to LTs for 1 d suggest that tissue conditions may favor NR-dependent NO production. Regardless of NR stimulation, a significant increase in NOS-like activity was observed in the roots, especially during the long-term treatment of plants with LT. These results indicate that diverse NO-producing routes, both reductive and oxidative, are activated in cucumber tissues at different stages of cold stress.

## 1. Introduction

Plants are constantly subjected to unfavorable environmental changes, which affect their metabolic and physiological processes. Unpredictable temperature fluctuations (especially temperature drops) are a common phenomenon in the temperate climate zone. Cold stress, which includes chilling (0–15 °C) and freezing (below 0 °C), is one of the most harmful abiotic factors limiting the growth and yield of many plants [1]. For this reason, it is important to understand how plants perceive low-temperature (LT) signals and how they respond to cold stress.

It is generally accepted that cell membrane remodeling and a change in its fluidity may initiate the perception of LT and simultaneously act as the first line of defense against this stress factor [2,3]. Changes in cell membrane viscosity and fluidity have been observed at LT stress. Moreover, membrane permeability increases, leading to cell dehydration [4]. These modulations trigger signal transduction, which initiates acclimation to cold stress. First of all, the expression of cold-regulated genes, encoding proteins that play an important role in protecting plants against LT, is modified [3,5,6,7]. During acclimation, plants enhance their cold tolerance by producing protective substances, such as soluble sugars, low-molecular-weight solutes, proline, and different groups of proteins [2].

The activation of signaling molecules is an essential element in plant defense mechanisms. They induce a cascade of reactions that enable plants to adapt to unfavorable conditions [8]. In recent years, nitric oxide (NO) has been shown to act as one of the important transmitters involved in signal transduction and plant responses to various abiotic stresses [9,10]. The coexistence of multiple reductive and oxidative NO-generating pathways has been confirmed in plants [11]. NO may be produced by a nitrite-dependent pathway, requiring enzymes containing molybdenum cofactor (MoCo), or through an L-arginine-dependent pathway (reviewed in [11,12,13,14,15]).

The reductive route involves the one-electron reduction of nitrite to NO, catalyzed by MoCo-using enzymes, among which nitrate reductase (NR) seems to be the most important [15]. The main function of cytoplasm-localized NR (cNR) is the two-electron reduction of nitrate to nitrite during nitrogen assimilation [16]. However, under specific conditions, NR may also transfer electrons to nitrite, resulting in NO production [17,18]. Moreover, a plasma membrane-bound form of NR (PM-NR) was found in plant roots. It is, together with membrane-bound nitrite-oxide reductase (Ni-NOR), probably part of the dual NO biosynthesis system [19,20,21]. PM-NR reduces nitrate to nitrite, which is then reduced to NO by Ni-NOR to generate NO in the apoplastic space [19]. Recently, in *Chlamydomonas reinhardtii*, a close relation between cNR and the amidoxime-reducing component (ARC), another MoCo-containing protein, was demonstrated during NO biosynthesis [22,23]. In this alga, the ARC was named nitric oxide-forming nitrite reductase (NOFNiR). It produces NO by reducing nitrite supplied by NR [23,24]. In higher plants, such interactions between ARC and NR have not been confirmed [25].

Under stress conditions, the acquisition of nitrate by plants and their reduction to nitrite are important steps not only in NO production, but also in the assimilation of nitrogen and the synthesis of building components. The regulation of NR plays a key role in controlling the balance between growth and adaptation [26]. In addition to NR, these processes involve both the uptake of nitrate by root cells via secondary symporters, driven by the proton gradient generated by the plasma membrane proton pump (H^+^-ATPase) [27], and the reduction of nitrite to ammonium ions, catalyzed by plastid nitrite reductase (NiR) [28]. Therefore, H^+^-ATPase and NiR participate in generating the current nitrate and nitrite pools (nitrite/nitrate ratio), which are essential for NO formation via reductive routes and further stages of nitrogen assimilation.

The oxidation of L-arginine is another possible pathway for NO production. In animals, this reaction is catalyzed by nitric oxide synthase (NOS). However, no homologous plant protein has been identified to date, so it has been suggested that this enzyme does not occur in plant cells [11]. On the other hand, some evidence allows us to assume that NOS-like activity leads to NO biosynthesis in higher plants [21,29,30,31].

Previous studies have shown that various pathways, both reductive (dependent on NR) and oxidative (dependent on NOS-like activity), may be involved in NO production in plants exposed to abiotic stresses. The aim of this study was to verify which of them are activated at different stages of cold stress in the roots of cucumber seedlings, exhibiting a significant increase in NO level. The main source of nitric oxide appears to be NR, and tungstate has been found to diminish NO generation in cucumber roots. For this reason, we examined the activity of this enzyme, analyzing both cytoplasmic and plasma membrane-bound NR, as well as the expression of *CsNR* and *CsARC* genes, encoding NR and its proposed partner protein, respectively, in plants treated with LTs. Moreover, nitrite reductase (NiR) activity and nitrate/nitrite content were determined. On the other hand, NOS-like activity was measured and the AET inhibitor was used to evaluate the role of the oxidative pathway in NO generation in the LT-stressed cucumber roots. To our knowledge, the presented research is the first to comprehensively demonstrate that different mechanisms are involved in increasing NO content in plant tissues subjected to LTs for short and long periods.

## 2. Results

### 2.1. Growth of Cucumber Roots Under LT Conditions

Cucumber seedlings were stressed with LT (10 °C) for short (1 d) or long (6 d) periods. The short-term LT treatment did not affect plant root growth. No significant differences were observed in root fresh (FW) and dry (DW) weight, water content (WC), or tissue amount of soluble protein between stressed and non-stressed plants (Table 1). In contrast, the long-term treatment of seedlings with LT caused a strong (80%) reduction in root FW and DW. The soluble protein content of stressed roots increased by approximately 20%, whereas WC did not change compared with the roots of non-stressed plants (Table 1).

### 2.2. Endogenous NO Level in Cucumber Roots

**Figure 1:**
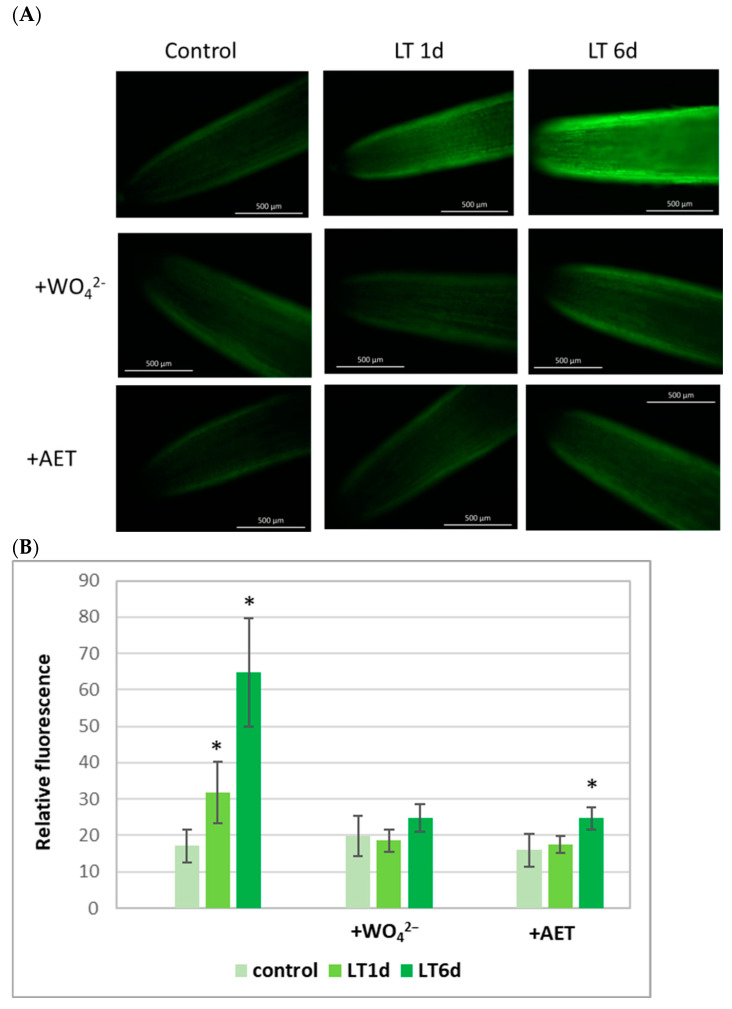
Bio-imaging of NO production (**A**) and mean DAF-2D-related fluorescence density (**B**) in root apical segments of cucumber seedlings not treated (control) and treated with LT for 1 day (LT 1 d) and 6 days (LT 6 d). Some sets of plants were incubated in the presence of an NR inhibitor (0.1 mM sodium tungstate; +WO_4_^2−^) or a mammalian NOS inhibitor (0.1 mM AET, +AET). Bio-imaging of NO generation was monitored by labeling with the NO-specific fluorescent dye, DAF-2D, and imaged using fluorescence microscopy. The images shown in (**A**) are representative for at least 3–5 roots for each treatment from 3 independent replications of the experiment. The asterisks in (**B**) indicate a difference in comparison to the control (Student’s *t*-test, *p* < 0.05).

LT led to an increase in NO content in cucumber roots. Both short and long LT exposure of plants caused significantly stronger fluorescence of 5,6-diaminofluorescein diacetate (DAF-2D), a NO-specific fluorescent dye, in roots than in non-stressed plants. An increase in DAF-2D fluorescence was observed in the bio-images of the root apical segments (Figure 1A) and relative fluorescence values (Figure 1B). To verify the involvement of reductive (dependent on NR) and/or oxidative (dependent on NOS-like activity) routes of NO biosynthesis in NO generation observed during LT stress, sodium tungstate (WO_4_^2−^), an inhibitor of nitrate reductase and other molybdoenzymes, and aminoethylthiorea bromide (AET), an inhibitor of animal NOS, were added to the nutrient solution. The production of NO was monitored, and the results are shown in Figure 1. Tungstate completely abolished the stimulation of NO production induced by both short and long LT treatments. DAF fluorescence measured in LT-exposed roots was similar to that in the control. At the same time, the addition of AET also diminished the DAF fluorescence observed in cucumber roots subjected to LT stress, suggesting a decrease in NO production (Figure 1A,B).

### 2.3. Activity of NR and Expression of CsNR Genes

The effect of tungstate on the NO level, observed in cucumber roots exposed to LT, suggests that NR may be involved in the enhanced production of NO. Therefore, the activities of both cNR and PM-NR were determined in roots treated with short- and long-term LT stress. According to our previous studies [21], total NR activity (NR_total_) in the presence of EDTA and actual NR activity (NR_act_) in the presence of Mg^2+^ were measured to express the level of dephospho-NR (dp-NR) and to estimate the NR activation state.

**Figure 2:**
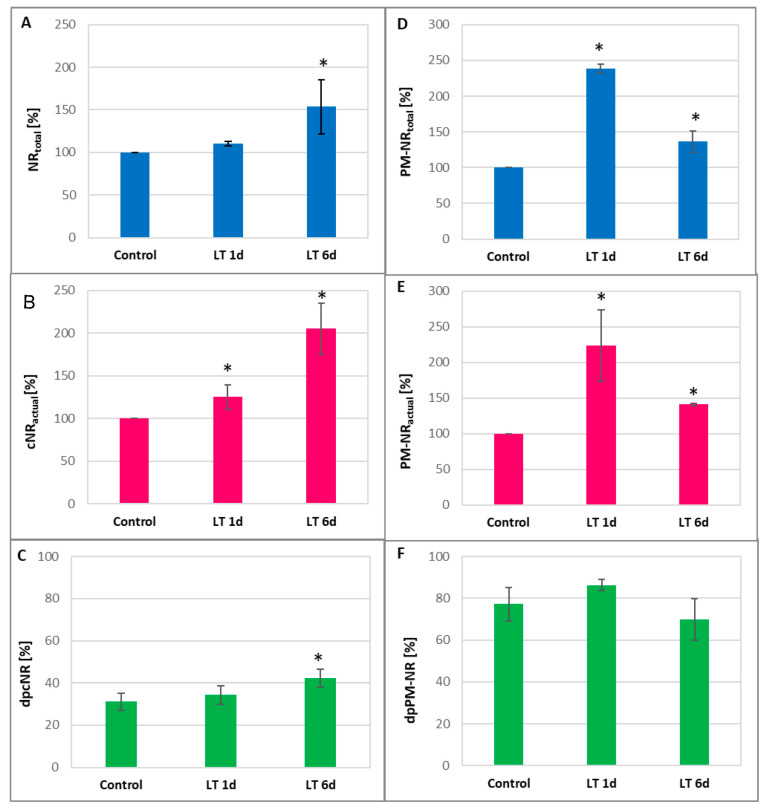
Total activity, actual activity, and enzyme activation state of cNR (**A**, **B** and **C**, respectively) and PM-NR (**D**, **E** and **F**, respectively) in roots of cucumber seedlings not treated (control) and treated with LT for 1 day (LT 1 d) and 6 days (LT 6 d). Further, 326.96 ± 40.1 (**A**) and 98.30 ± 15.05 (**B**) nmol NO_2_^−^ × g FW^−1^ × h^−1^ were used as 100% for cNR. Additionally, 223.22 ± 59.16 (**D**) and 155.01 ± 35.01 (**E**) nmol NO_2_^−^ × mg^−1^ protein × h^−1^ were used as 100% for PM-NR. All results are means ± SDs of 3 replications of 4 independent experiments. The asterisks indicate significant differences in comparison to the control (Student’s *t*-test, *p* < 0.05).

A significant increase in NR activity was observed after the exposure of plants to LT. The cNR activity showed the highest level in roots treated with LT for 6 d. cNR_total_ and cNR_act_ reached about 150% and 200% of the control value, respectively (Figure 2A,B). This led to an increase in the cNR activation state in roots subjected to long-term LT stress, suggesting that post-translational enzyme dephosphorylation occurred (Figure 2C). In contrast, PM-NR activity was most stimulated in the roots of seedlings exposed to LT for a short time. After 1 d of LT treatment, PM-NR_total_ achieved approximately 245%, whereas PM-NR_act_ achieved 225% compared to the control (Figure 2D,E). The results indicate that the PM-NR activation state remained unchanged under LT stress conditions (Figure 2F).

**Figure 3:**
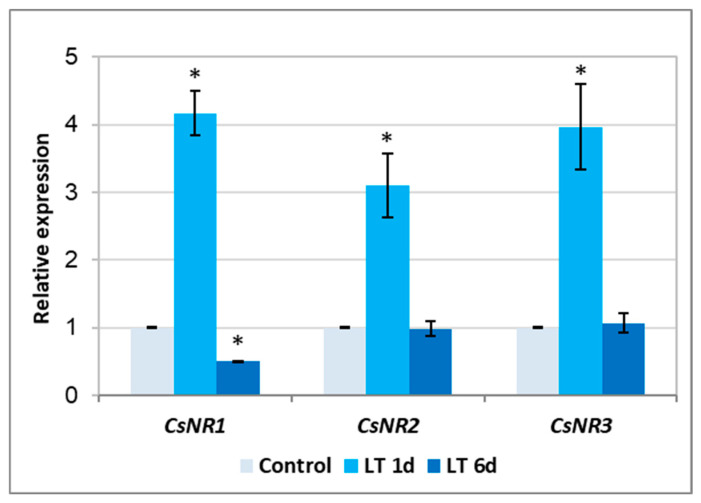
Relative expression of CsNR1-3 genes in roots of cucumber seedlings not treated (control) and treated with LT for 1 day (LT 1 d) and 6 days (LT 6 d). Expression was determined using real-time PCR. All results were normalized to the reference expression of CsTIP41 encoding the TIP41-like protein. The values presented are means ± SDs from 3 replicates of 3 independent experiments. The asterisks indicate significant differences compared to the control (Student’s *t*-test, *p* < 0.05).

The NR is encoded by three genes in the cucumber genome [32]. To verify the possibility that the enzyme activity is regulated by LT at the gene expression level, the transcription of CsNR genes was determined. It was found that the short exposure of plants to LT significantly activated (approximately 3–4 times) the expression of all analyzed genes. However, the 6-day LT treatment did not affect the expression of CsNR2 and CsNR3 isoforms and, at the same time, inhibited the transcription of CsNR1 (Figure 3).

### 2.4. Activity of Nitrite Reductase and Nitrate/Nitrite Content

**Figure 4:**
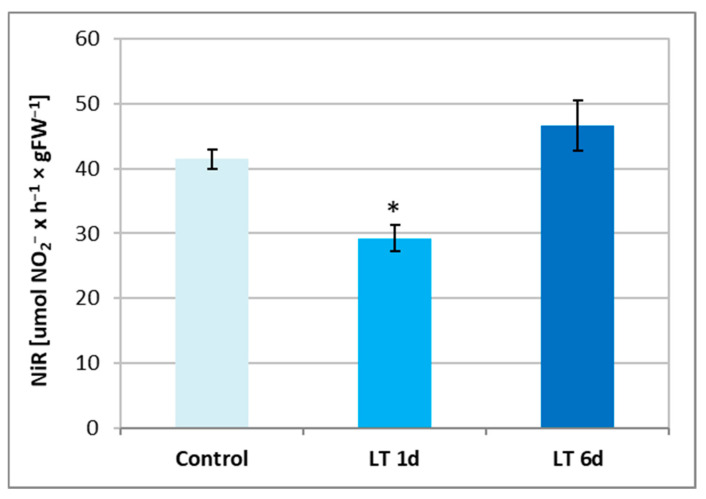
Nitrite reductase (NiR) activity in roots of cucumber seedlings not treated (control) and treated with LT for 1 day (LT 1 d) and 6 days (LT 6 d). The data presented are the means of 3 replications of 5 independent experiments. Error bars represent SDs. Asterisks indicate significant differences compared to the control (Student’s *t*-test, *p* < 0.05).

To evaluate whether tissue conditions can promote NO generation via NR activation, the levels of nitrate and nitrite ions, as well as nitrite reductase (NiR) activity, were analyzed in cucumber roots treated with LTs. NiR activity was diminished only in plants exposed to LTs for a short time. The observed inhibition was approximately 25%, relative to that of the control (Figure 4).

Moreover, the nitrate level was visibly reduced in roots after 1-day LT treatment, reaching approximately 55% in comparison to the control. No change was observed in plants exposed to LT for 6 days (Table 2). On the other hand, the nitrite content increased in roots after longer exposure to LT, reaching above 140% of the control value, and remained unchanged in roots treated for 1 d (Table 2). The results showed that the nitrite/nitrate ratio was significantly higher in plants exposed to LT. It increased more than twice in cucumber roots exposed to short-term stress and was enhanced by 34% in roots subjected to long-term stress compared to the control (Table 2). Therefore, a higher NO_2_^−^/NO_3_^−^ ratio and a decrease in NiR activity may be beneficial for the NR-dependent production of NO under LT stress.

### 2.5. NOS-like Activity

**Figure 5:**
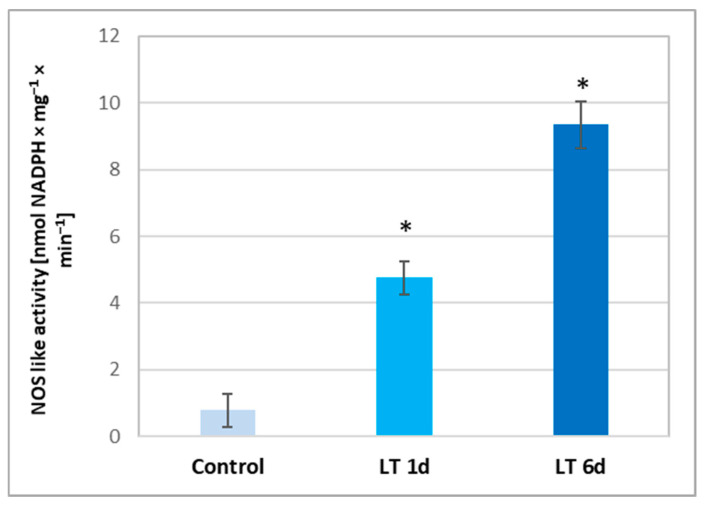
NOS-like activity measured as arginine-dependent NADPH oxidation in extracts prepared from roots of cucumber seedlings not treated (control) and treated with LT for 1 d (LT 1 d) and 6 d (LT 6 d). Data presented are means of 3 replicates of 3 independent experiments, with error bars representing the SD. Asterisks indicate significant differences compared to the control (Student’s *t*-test, *p* < 0.05).

In addition to the reductive pathway (related to NR activity) that participates in NO production in plant cells, the oxidative route related to NOS-like activity (similar to mammalian NOS) may be involved in this process. As we have shown, using AET, a well-known NOS inhibitor, this pathway can be activated in seedlings grown under LT stress. Therefore, in the next step, the NOS-like activity was measured in cucumber roots. It was demonstrated that LT evoked a significant increase in the NOS-like activity level, which depended on the time of plant exposure. Activity increased approximately 5-fold and 10-fold in the tissues of roots exposed to LT for 1 and 6 d, respectively (Figure 5). These results confirmed that the oxidative pathway is involved in NO generation in cucumber under LT stress.

### 2.6. Expression of ARC Gene

**Figure 6:**
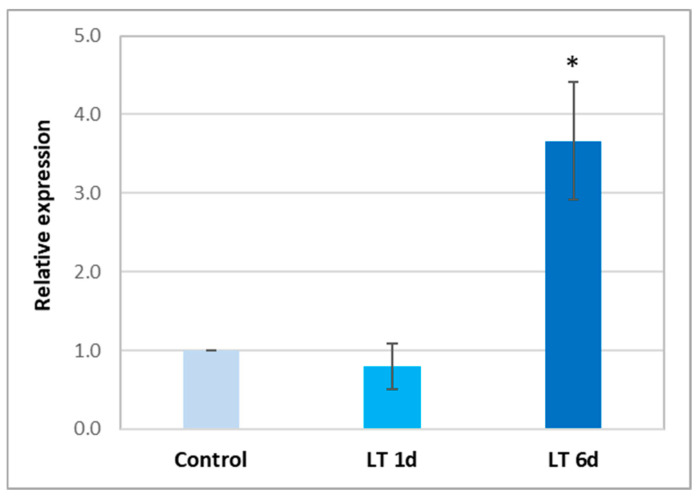
Relative expression of *CsARC* in the roots of cucumber seedlings not treated (control) and treated with LT for 1 d (LT 1 d) and 6 d (LT 6 d). Expression was determined by real-time PCR. The results were normalized to the reference expression of *CsCACS*, which encodes a clathrin adaptor complex subunit. Presented values are means ± SDs of 3 replicates of 3 independent experiments. Asterisks indicate significant differences compared to the control (Student’s *t*-test, *p* < 0.05).

NR activity may be related to another Mo-containing protein, ARC, which can directly reduce nitrite to NO. The cucumber genome was searched using BLASTn (https://blast.ncbi.nlm.nih.gov/) to identify nucleotide sequences homologous to the *Arabidopsis AtARC1* and *AtARC2*. One sequence, with a total length of 888 bp and the highest homology to *AtARC1* (67.45%) was identified (Figures S1–S4). Therefore, we analyzed the expression level of the newly identified *CsARC* gene in cucumber roots. Long-term exposure to LT causes an approximately 3.5-fold increase in ARC transcription (Figure 6). These results are consistent with the increase in cNR activity observed in roots under long-term LT treatment (Figure 2A,B).

## 3. Discussion

NO plays an important role in the adaptation of plants to unfavorable environmental conditions, including cold stress [9]. NO, together with other gasotransmitters, acts as a signal activating defense mechanisms in plant cells [8]. When plants are exposed to LT, increased NO production is observed in tissues including *Arabidopsis* [33], cucumber [34], the subtropical bromeliad *Nidularium minutum* [35] and legumes [36]. In cucumber roots, increased NO levels occurred in both seedlings subjected to short- and long-term cold stress (Figure 1). Different sources of NO generation function in higher plants. For this reason, we decided to explain whether diverse routes of NO production might be induced in response to LT, depending on the duration of stress. There are two alternative pathways for NO production in plants: oxidative (L-arginine-dependent) and reductive (nitrite-dependent) [15]. Using specific inhibitors, we showed that both mechanisms were involved in NO generation in cucumber roots exposed to LT (Figure 1). Thus, it was interesting to determine the role of individual pathways in generating the NO signal at different stages of cold stress.

The oxidative pathway is related to the enzymatic activity of NOS. In this process, L-arginine first interacts with molecular oxygen, creating N-hydroxy-L-arginine, which is in turn converted to citrulline, releasing NO [15]. This reaction and the NOS enzyme have been well-characterized in animals. Recently, the possible presence of NOS proteins in higher plants has been increasingly questioned since homologous proteins/genes have not yet been identified, despite the analysis of a large number of plant transcriptomes [11,30]. On the other hand, the protein with 45% homology to mammalian NOS sequences was identified in photosynthetic unicellular algae of the *Ostreococcus* family [30,37]. It appears that during evolution, plants lost genes similar to the mammalian NOS genes. However, it should be emphasized that numerous studies have confirmed that the use of inhibitors of mammalian NOS activity (e.g., AET) leads to the inhibition of NO production in the tissues of various species of higher plants [21,29,30,31]. On this basis, it has been suggested that some polypeptides, containing active redox domains that can catalyze the reactions of arginine-dependent NO formation, function in the cells of higher plants [15]. Therefore, the goal of many research centers is to identify these polypeptides. NOS-like activity has also been observed in cucumber seedlings [38]. Moreover, we showed that this activity increased significantly when the plants were exposed to LT (Figure 5). NOS-like activity was significantly higher in roots already at the initial stages of cold stress, i.e., in seedlings growing under short-term stress conditions, compared to control plants. However, during long-term exposure to LT, stimulation of enzyme activity doubled compared to that in plants treated with cold for a short period (Figure 5). These results suggest that the oxidative route of NO generation is activated in cucumber roots under LT conditions, but it plays a more important role during long-term exposure.

The reductive pathway for NO generation is related to the reduction of the nitrite formed during nitrate assimilation [39]. Nitrate is an important nutrient in plants [40]. For this reason, any modifications in its uptake and assimilation affect many processes, including growth (formation of building components) and signal transduction (participation in the production of NO). LT is known to strongly affect plant growth processes [1]. During the short, one-day cold period, we did not observe any significant changes in the growth parameters of the cucumber seedlings. However, an inhibitory effect of cold was visible in the roots of plants treated with LT for a longer time (Table 1). The growth of plant organs depends on the uptake of nutrients and, to a large extent, on the activity of the plasma membrane proton pump (PM H^+^-ATPase). This enzyme is involved in the process of acidic cell wall growth, as well as in generating a proton gradient across the plasma membrane used by secondary transporters to co-transport ions (e.g., symporters nitrate/protons) [41]. Our research showed that the level of nitrate in root tissues changed depending on the LT treatment of plants. In plants treated with a short period of cold (1 day), the nitrate content decreased significantly and was only half of the value determined in the tissues of control plants. However, under longer cold treatment, the nitrate level returned to that observed in the tissues of the control plants (Table 2). Plants take up nitrate from the soil solution via an energy-dependent system [42]. Nitrate uptake usually involves H^+^/nitrate cotransport. This mechanism of nitrate uptake was first demonstrated in Lemna [43]. Since the nitrate uptake by plants is accompanied by the alkalization of the external environment, it is believed that this transporter functions with a 2:1 stoichiometry for H^+^/NO_3_^−^ [28,44]. This means that the uptake of nitrate requires the prior transfer of protons outside the plasma membrane. The only enzyme protein present in the plasma membrane that can transfer protons from the cytoplasm to the apoplast is the plasma membrane proton pump [41]. Our previous study [34] showed that treating plants (cucumber seedlings) with LT for one day inhibited the activity of PM H^+^-ATPase. In contrast, under longer cold stress (six days), this activity increased. It can be concluded that the reduced nitrate content observed in plants after a one-day exposure to LT may result, among other things, from the inhibition of the PM proton pump.

On the other hand, the level of nitrate in tissues is regulated by the activity of nitrate reductase. Nitrate taken up by the roots is reduced through nitrite to ammonium ions. The first step in this reduction is catalyzed by the cytosolic enzyme, nitrate reductase [44]. In addition to its cytosolic location (cNR), the enzyme is also found near the plasma membrane, in PM-NR form [21,28]. In the present study, NR activity increased under both short-term and long-term LT stress (Figure 2). It was observed, however, that depending on the duration of LT exposure, different changes in NR activity, related to its location in the cell, occurred in roots. Under short-term cold stress, a clear increase in the activity of PM-NR was shown, while under long-term cold stress, a significant increase in cNR activity was demonstrated (Figure 2). It is well known that NR activity is subjected to comprehensive and tight regulation at both the genetic and post-translational levels. This enables precise modulation of the enzyme activity and allows plants to adjust the intensity of nitrogen assimilation and NO production to current conditions and environmental stimuli [23,45,46]. On the other hand, by analyzing the functional properties of NIA1 and NIA2, two isoforms of *A. thaliana* NR, it was found that the first protein is mainly involved in NO production while the second one is involved in nitrate reduction [47]. An increase in the relative abundance of NR-specific mRNA was observed in cucumber plants during the 24 h incubation of plants at 5 °C [48]. We have shown that the expression of all three genes encoding nitrate reductase in cucumber was upregulated only in roots exposed to LT for a short period (Figure 3). It corresponded with the higher PM-NR activity observed in roots under these conditions (Figure 2). This suggests that another regulatory mechanism is responsible for changes in NR activity, especially under long-term cold conditions.

The post-translational regulation of NR activity mainly involves the rapid and reversible phosphorylation/dephosphorylation of enzyme proteins. Phosphorylated NR protein (pNR), in the presence of Mg^2+^ ions, is recognized by the inhibitor protein 14-3-3, which leads to the reversible inhibition of the enzyme activity [45,46]. The dephosphorylation of NR or removal of divalent cations from the reaction medium enables the breakdown of the inactive pNR-14-3-3 complex and the recovery of the catalytic activity of NR. By determining the actual NR activity and total NR activity, it is possible to estimate the level of the dephosphorylated active form of NR (dp-NR), also known as the activation state of NR. Changes in dp-NR level indicate changes in the degree of NR phosphorylation, and thus post-translational modifications of NR which modulate its activity [9,21]. A significant increase in the cytoplasmic dp-NR level was observed in the roots of plants subjected to long-term LT (Figure 2C) and correlated with a concomitant marked increase in the catalytic activity of cNR (Figure 2B), indicating that post-translational modifications, in addition to changes at the genetic level, affect NR activity under cold stress. Liu et al. [49] demonstrated that in older cucumber plants (with two fully expanded leaves), total NR activity decreased in roots treated at 8 °C for 5 h without any changes in actual NR activity. Simultaneously, the downregulation of *CsNR1* and *CsNR2* transcription and the upregulation of *CsNR3* transcription were observed. The Expression of genes encoding NR in *Brassica juncea*, *BjNR1* and *BjNR2*, was also inhibited in seedlings subjected to low temperatures (4 °C) for 1 and 24 h [50].

Similarly to our results, stimulation of NR activity was observed in the bromeliad *Nidularium minutum* exposed to 10 °C for 72 h [35] and *Medicago* during exposure to 5 °C for 1 to 21 days [36]. Yaneva et al. [51] found that, in the leaves of winter wheat, LT (2 h at 4 °C) increased actual NR activity but did not change total NR activity. This increase correlated with enhanced gene expression level, but not with the protein level of NR. Using specific protein kinase and phosphatase inhibitors, the authors indicated that dephosphorylation acts as a mechanism for NR activation under LT conditions [51].

Some studies indicate that, during LT stress, the activation of the nitrite-dependent pathway leads to NO production by NR [36,48]. The role of NR in the acclimation of *Arabidopsis* to LT was confirmed by Zhao et al. [33]. Mutants with deletions of both nitrate reductase genes (*nia1nia2*) were less tolerant to low temperature than wild-type plants. Furthermore, using both a NR inhibitor as well as a NO scavenger, it was shown that NR-dependent NO production plays an important role in cold acclimation by the stimulation of proline accumulation [33].

By reducing nitrate, NR contributes to the production of nitrite, which immediately enters the plastids. They are then reduced to ammonium ions by nitrite reductase (NiR) [28]. In our study, we did not observe any significant changes in nitrite levels in plant roots as a result of one-day cold exposure, but, in contrast, there was a noticeable increase in the nitrite content in tissues during long-term cold exposure (Table 2). This may be related to the increased proton pump activity observed in cucumber roots under long-term LT stress [34]. Under such conditions, the enzyme can generate a larger proton gradient, which is then used by secondary nitrate transporters to uptake increased amounts of nitrate, which is reduced to nitrite by enhanced NR activity. Moreover, NiR activity was inhibited in cucumber roots under short-term (1 day) cold conditions (Figure 4), which additionally favored the accumulation of nitrite in the tissue. Liu et al. [49] found that, in older cucumber plants, NiR activity and the *CsNiR* transcript remained at the same level in the roots exposed to 8 °C and 26 °C for 5 h. Changes in the nitrate and nitrite levels as well as NR and NiR activities affect the nitrate-to-nitrite ratio (Table 2). We showed that cold stress promoted an increase in the NO_2_^−^/NO_3_^−^ ratio in cucumber roots. It increased by more than 100% and 50% in plants treated with LT for short and long periods, respectively. The NO_2_^−^/NO_3_^−^ ratio seems to be an important factor in favoring NR-dependent NO production [21]. The available literature data indicate that NO production is intensified under conditions in which high levels of nitrite are present in the cytosol and, at the same time, an inhibition of plastid NiR activity and relatively low levels of nitrate are found [21,23,52]. These changes may have contributed to the observed increase in NO production in cucumber seedlings exposed to LT. This pathway appears to be particularly important for NO generation during short-term cold stress, since the inhibition of NiR activity (Figure 4) and the highest nitrite/nitrate ratio (Table 2) were found under these conditions. In *Arabidopsis*, NiR has been proposed to be an essential element involved in the regulation of nitrogen assimilation and NO homeostasis during plant growth and adaptation to stress conditions [53].

In higher plants, nitrate reductase is considered the main enzyme involved in the reductive pathway, leading to NO production [15]. However, in the single-celled green alga *Chlamydomonas reinhardtii*, the presence of another important protein, ARC, which may participate in NO generation via a reduction pathway, was demonstrated. This protein, also known as NOFNiR, closely interacts with cytoplasmic NR to produce NO [22]. ARC reduces nitrite resulting from nitrate reduction catalyzed by NR [23]. In higher plants, the ARC protein has been characterized only in *Arabidopsis* [25]. The presence of two genes encoding ARC in *Arabidopsis*, *AtARC1* and *AtARC2*, has been demonstrated. Both ARC isoforms are Mo-dependent enzymes capable of catalyzing the reduction of N-hydroxylated compounds [25]. Therefore, it can be assumed that, in general, in higher plants, ARC proteins participate in the production of NO via the reduction pathway. However, no functional connection between ARC and NR has been demonstrated as in *Chlamydomonas*, and the functions of these proteins in plants have not been elucidated [25]. This is still an open issue that requires intensive research on various species of higher plants. We searched the cucumber genome and found one gene encoding ARC, with high homology to *AtARC1* and *AtARC2* (Figures S1–S4). We named it *CsARC*. Interestingly, the expression of this gene changed significantly in the roots of cucumber plants exposed to low temperatures for a long time, reaching a level almost four times higher than in control seedlings (Figure 6). In algae, it has been shown that this protein participates, together with cNR, in the production of NO [22]. Consistent with this, our study showed a relationship between NR and ARC after six days of cold exposure, i.e., under conditions during which a significant increase in both cytosolic NR activity and *CsARC* expression was observed (Figure 2A,B). Therefore, it can be speculated that the production of NO by cytosolic NR is supported by ARC in cucumber plants under long-term LT stress. However, this point of view requires further investigation.

## 4. Materials and Methods

### 4.1. Plant Material, Growth Conditions, and Measurements of Growth Parameters

All experiments were conducted on 7-day-old seedlings of cucumber (*Cucumis sativus* L. cv. Wisconsin). The seeds were sterilized in 3% H_2_O_2_ and, after rinsing, were germinated at 25 °C in darkness for 48 h. After germination, they were transferred to a nutrient medium composed of 1.7 mM KNO_3_, 1.7 mM Ca(NO_3_)_2_, 0.33 mM KH_2_PO_4_, 0.33 mM MgSO_4_, 25 µM ferric citrate, 3.33 µM MnSO_4_, 1.7 µM H_3_BO_3_, 0.3 µM CuSO_4_, 0.003 µM ZnSO_4_, and 0.017 µM Na_2_MoO_4_ (pH 6.2). The plants were grown hydroponically in a 16 h photoperiod with light intensity of 180 µmol m^−2^ s^−1^ at 25 °C/22 °C during day/night (control conditions). The seedlings were treated with low temperatures (LT, 10 °C) for a short period (1 day, 1 d) or long period (6 days, 6 d) before the experiments. All analyses were performed using roots collected 4 h after the start of the day period. Some basic growth parameters such as fresh (FW) and dry weight (DW), root water content (WC), and amount of total soluble proteins were determined. Fresh roots were weighed (FW) and then dried at 80 °C for 48 h for DW evaluation. The percentage ratio between FW and DW indicated WC in the roots. The total soluble proteins were extracted from fresh roots at 4 °C for 30 min in 50 mM Tris-HCl buffer (pH 7.5) with 5 mM DTT and 2 mM PMSF. After centrifugation, the level of proteins was measured according to the Bradford method [54] with BSA as the standard. All reagents used to prepare the nutrient solutions were sourced from Chempur (Piekary Śląskie, Poland). Regents used for endogenous NO detection, isolation of the plasma membrane fraction, and enzyme activity measurement were purchased from Sigma-Aldrich (St. Louis, MO, USA).

### 4.2. Detection of Endogenous NO

The level of NO in root tissues was assayed using the fluorescent NO indicator dye, 5,6-diaminofluorescein diacetate (DAF-2D). Excised roots were briefly incubated in 20 mM Hepes-KOH, pH 7.4, containing 10 µM DAF-2D for 10 min at room temperature in the dark [9]. To remove excess fluorophore from the surface, the roots were washed for 15 min in fresh Hepes-KOH buffer, renewed twice. NO-specific fluorescence was detected with the fluorescent microscope Zeiss Axio Image M2 using a Tag-YFP filter with emissions of 524 nm and unchanged parameters for every measurement. The intensity of green fluorescence was analyzed using Corel Photopaint SE software (version 18.2.0.840) and expressed as the average number of pixels in the green channel on a scale ranging from 0 to 225.

### 4.3. Preparation of Cytosol and Isolation of Plasma Membrane Fraction

The roots of cucumber seedlings were cut off, rinsed in distilled water, and homogenized in chilled mortar with the addition of 1 mM PVPP and 25 mM BTP-MES (pH 7.5) containing 330 mM sorbitol, 5 mM EDTA, 5 mM DTT, 0.5 mM PMSF, and 0.2% BSA. The homogenate was centrifuged for 10 min at 18,000× *g* at 4 °C and the supernatant was used for cytoplasmic NR (cNR) activity determination and for isolation of the plasma membrane (PM) fraction by a two-phase system according to Janicka et al. [55] with some modifications described by Reda et al. [21]. The high purity of the obtained PM fractions was confirmed by measuring the activity of two cytosol marker enzymes, alcohol dehydrogenase (ADH), and phosphoenolpyruvate carboxylase (PEPC) according to Chung and Ferl [56] and Spalding and Edwards [57], respectively. The amount of protein in PM fractions was determined using BSA as a protein standard according to the method of Bradford [54].

### 4.4. Determination of Nitrate Reductase and Nitrite Reductase Activities

Nitrate reductase (NR, EC1.7.1.1.) activity was measured in cytosol (cNR) and PM fraction (PM-NR) obtained from cucumber roots. The total (NRtot) and actual (NRact) NR activities were determined in the absence or presence of Mg^2+^ ions according to Kasier and Huber [58], with further modifications previously described by Reda et al. [21]. The NRact/NRtot activity ratio was calculated as a percentage [9]. Nitrite reductase (NiR, EC1.7.7.1.) activity was determined colorimetrically according to Orea et al. [59] with some modifications described by Reda et al. [21].

### 4.5. NOS-like Activity Assay

NOS-like activity was performed according to Sun et al. [60]. Root tissue (1 g) was homogenized in a chilled mortar with the addition of 1 mM PVPP and extraction buffer (100 mM Hepes-KOH, pH7.5) containing 1 mM EDTA, 5 mM DTT, 0.5 mM PMSF, 10% glycerol (*v*/*v*), 0.1% Triton X-100 (*v*/*v*), and 20 µM FAD. The homogenate was centrifuged for 20 min at 13,000× *g* at 4 °C, and the supernatant was used for NOS-like activity determination. The reaction mixture (1 mL) was composed of 100 mM phosphate buffer (pH 7.0), 2 mM MgCl_2_, 0.3 mM CaCl_2_, 4 µM tetrahydrobiopterin, 1 µM FAD, 1 µM FMN, 0.2 mM DTT, 0.2 mM NADPH, 1 mM L-Arginine, and 200 µL of tissue extract. Consumption of NADPH was measured spectrophotometrically as the decrease in absorbance at 340 nm, and activity was calculated using the NADPH extinction coefficient (ε = 6.22 mM^−1^ cm^−1^).

### 4.6. RNA Isolation, cDNA Synthesis, and Real-Time PCR Analysis

Total RNA was isolated from root tissue with TriReagent (Sigma-Aldrich, St. Louis, MO, USA) according to the manufacturer’s instructions. The purity and amount of RNA preparations were determined spectrophotometrically using NanoDrop ND-1000 (Thermo Fisher Scientific, Waltham, MA, USA). Samples displaying the 260/280 and 230/260 ratios between 1.8 and 2.0 were used for further analysis. After treatment with RNase-free DNase I (Thermo Fisher Scientific, Waltham, MA, USA), the purified RNA samples were used as a template for first strand cDNA synthesis with the High-Capacity cDNA Reverse Transcription Kit (Applied Biosystems, Waltham, MA, USA) according to the manufacturer’s instructions. Synthesized cDNA was used for real-time PCR performed with a LightCycler 480 system (Roche, Basel, Switzerland) and the Real-Time 2xPCR Master Mix SYBR kit (A&A Biotechnology, Gdańsk, Poland). The amplification conditions were as follows: 30 s at 95 °C; 35–40 cycles of 10 s at 95 °C; 10 s at 56 °C (for NR genes) and 60 °C (for ARC gene); 12 s at 72 °C; and final melting for 15 s at 65 °C. Melting curve analysis was performed to confirm the specificity of the amplicons. A dilution of the samples with the lowest crossing point (Cp) was used as a standard curve with an amplification efficiency of around 2. The expression of genes encoding the cucumber tonoplast intrinsic protein, CsTIP41, and the clathrin adaptor complex subunit, CsCACS, were used as the internal standards [61]. The sequences of primers specific to amplified genes were designed using LightCycler ProbeDesign software 2 (Roche, Basel, Switzerland) and are listed in Table 3.

### 4.7. Endogenous Nitrate and Nitrite Level Measurement

Levels of endogenous nitrate and nitrite ions were determined in Milli-Q water (Merck Millipore, Darmstadt, Germany) extracts prepared from root tissue (100 mg) powdered in liquid nitrogen according to Reda [62]. The contents of NO_3_^−^ and NO_2_^−^ in the extracts were measured using HPLC system (LKB, Vienna, Austria) with an ion exchange column Sphere-Image 80-5 SAX (Knauer, Berlin, Germany) with 30 mM NaH_2_PO_4_-H_3_PO_4_, pH 3.0, as the liquid phase, and 0.5 mM KNO_3_ and 0.5 mM NaNO_2_ as standards.

### 4.8. Statistics

The data presented in the figures and tables are means ± SDs (standard deviations) from at least three replications performed in three or more independent experiments, as indicated in the legends. The quantitative real-time PCR data were analyzed using LightCycler software 4.1 (Roche). Statistical analysis of the results was carried out using Student’s *t*-test (Excel) and *p*-values ≤ 0.05 were accepted as significant.

## 5. Conclusions

The exposure of cucumber seedlings to LT for both short (1 d) and long (6 d) periods leads to increased root NO production. Under short-term cold conditions, NO formation is mainly mediated by PM-NR, which is an element of the reductive pathway. This action may be supported by the high nitrite/nitrate ratio associated with inhibited NiR activity. Under long-term cold conditions, enhanced NO production results from the activation of both the oxidative pathway, i.e., increased NOS-like activity, and the reductive pathway, related to the activity of cNR, and probably its interaction with the ARC protein. Although both oxidative and reduction routes participate in the generation of NO in cucumber roots under LT conditions, different mechanisms are involved in this process depending on the duration of the stress factor.

It is well known that the perception of environmental stimuli including LT takes place at the plasma membrane. Cold exposure was found to induce both structural and qualitative changes in the plasma membrane. Therefore, it can be suggested that in the first stage of the reaction of cucumber roots to LT, PM-bound NR is activated. This leads to an increase in NO production and the induction of signaling pathways that trigger defense mechanisms by inducing the expression of appropriate genes, including those encoding NR. Consequently, increased cNR activity is observed under prolonged cold stress. During longer cold stress, the plant adapts to the changed conditions, modifies its metabolism, and activates many defense mechanisms. Therefore, in addition to the reductive pathway, the oxidative route can be induced, and a higher NO level is observed in the roots compared to plants treated with LT for a short time.

We confirmed, as expected, that both NO generation routes function in cucumber roots under LT conditions, with the reductive pathway being activated during short-term stress and the oxidative pathway being additionally induced during long-term stress.

## Figures and Tables

**Table 1:** Growth parameters in roots of cucumber seedlings under LT stress.

	Control	LT 1 d	LT 6 d
FW [g]	0.141 ± 0.011	0.140 ± 0.007	0.030 ± 0.006 *
DW [g]	0.0063 ± 0.0006	0.0064 ± 0.0006	0.0016 ± 0.0002 *
WC [%]	95.64 ± 0.14	95.45 ± 0.27	94.85 ± 0.19
Total soluble protein [mg × g FW^−1^]	1.53 ± 0.13	1.42 ± 0.09	1.87 ± 0.07 *

Fresh weight (FW), dry weight (DW), water content (WC), and total soluble protein level in roots of cucumber seedlings treated with LT for 1 day (LT 1 d) and 6 days (LT 6 d). The presented values are means ± SDs of 3 independent experiments, and each experiment included 20 seedlings per treatment. Statistically significant differences (Student’s *t*-test) between the control and treatments are marked as * (*p* < 0.05).

**Table 2:** Nitrate and nitrite levels and NO_2_^−^/NO_3_^−^ ratio in cucumber roots under short (1 d) and long (6 d) LT stress.

	Control	LT 1 d	LT 6 d
NO_2_^−^	7.56 ± 0.74	8.33 ± 0.71	10.86 ± 1.24 *
NO_3_^−^	32.47 ± 1.97	17.89 ± 3.69 *	33.86 ± 3.20
NO_2_^−^/NO_3_^−^	0.23 ± 0.02	0.48 ± 0.14 *	0.31 ± 0.01 *

Levels of nitrate and nitrite ions in cucumber roots were measured in water tissue extracts by HPLC as described in the Materials and Methods Section. Presented values (µmol × g FW^−1^) are means of 3 replications of 3 independent experiments ± SD. Statistically significant differences (Student’s *t*-test) between the control and treatments are marked with * (*p* < 0.05).

**Table 3:** The list of primers used in qRT-PCR analysis.

Gene	Forward Primer (5′–3′)	Reverse Primer (5′–3′)	Temp of Anniling	Ref.
*CsNR1* *CsNR2* *CsNR3*	GGACGGTAGAGTAAAGAAGGC CGACTCCTCCTCCAACTCC TCCAATGGCGACTGCTG	TATCCCTTTTACTCCATTCA CCACTTCCATGTTGTCCAA CATCATCATCAATAAGGAGCGG	56 °C	[32]
*CsARC*	TTCTTGTTGATGGCTGCGA	AGTTTCATTCAGCTCAGGTC	60 °C	
*CsCACS* *CsTIP41*	TGGGAAGATTCTTATGAAGTGC CAACAGGTGATATTGGATTATGATTATAC	CTCGTCAAATTTACACATTGGT GCCAGCTCATCCTCATATAAG	60 °C 56 °C	[61]

## Data Availability

The data presented are available in this manuscript.

## Supplementary Materials

The following supporting information can be downloaded at https://www.mdpi.com/article/10.3390/ijms26041599/s1.
