# Supplementary material for: Regulation of NO-Generating System Activity in Cucumber Root Response to Cold
Source: Int J Mol Sci. 2025 Feb 13;26(4):1599. doi: 10.3390/ijms26041599 (PMC11855582; doi:10.3390/ijms26041599)
Supplement: Supplementary file 1 [file ijms-26-01599-s001.zip › ijms-3465468-supplementary.pdf]

Supplementary

Regulation of NO-Generating System Activity in Cucumber Root Response to Cold

Małgorzata Reda, Katarzyna Kabała, Jan Stanisławski, Kacper Szczepski and Małgorzata Janicka *

Department of Plant Molecular Physiology, Faculty of Biological Sciences, University of Wrocław, Kanonia 6/8, 50-328 Wrocław, Poland; malgorzata.reda@uwr.edu.pl (M.R.); katarzyna.kabala@uwr.edu.pl (K.K.); janski9999@gmail.com (J.S.); kacper.szczepski@kaust.edu.sa (K.S.)

* Correspondence: malgorzata.janicka@uwr.edu.pl

FGENESH 2.6 Prediction of potential genes in Dicot_arab genomic DNA
Seq name: ACHR02000006.1:28952665-28959378 Cucumis sativus cultivar 9930 Chr6, whole gen
Length of sequence: 6714
Number of predicted genes 1: in +chain 0, in -chain 1.
Number of predicted exons 8: in +chain 0, in -chain 8.
Positions of predicted genes and exons: Variant 1 from 1, Score:50.294348

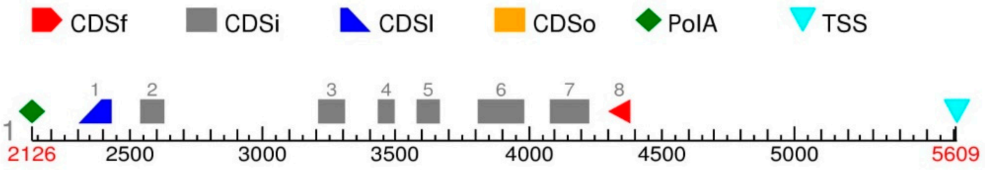


```
1 -      PolA      2126             1.06
1 -    1 CDSl      2301 -     2426 16.42      2301 -     2426   126
1 -    2 CDSi      2533 -     2625  9.76      2533 -     2625    93
1 -    3 CDSi      3203 -     3305 12.18      3203 -     3304   102
1 -    4 CDSi      3428 -     3492 -3.71      3430 -     3492    63
1 -    5 CDSi      3574 -     3662  7.91      3574 -     3660    87
1 -    6 CDSi      3804 -     3980 20.31      3805 -     3978   174
1 -    7 CDSi      4076 -     4225  4.93      4077 -     4223   147
1 -    8 CDSf      4295 -     4379  2.01      4296 -     4379    84
1 -      TSS       5609            -8.54

Predicted protein(s):
>FGENESH:[mRNA] 1 8 exon (s) 2301 - 4379 888 bp, chain -
ATGGCGGCTGTGTCTGCAATCTTCATTTACCCAATCAAATCATGTCGCGGTATTTCAGTT
CCCCAAGCACCTCTTACCCCTACTGGATTTCGATGGGACCGTCAATGGTTGGTTGTCAAC
TCTAAAGGAAGGGCTTACACTCAGAGAGTCGAGCCTAAGCTTGCTCTCGTCCAGGTCGAC
CTCCCAAACGAAGCTTTCTTTGATGACTGGAGCCCTTCTAAATCCTCTTTTCTCGTCATT
AAAGCTCCTGGGATGGATGTCTTAAAGGTTCCCCTTACTCAACCTCCGTATAATGCTGAC
GGTGTCTCTGTCTGGGAATGGTCTGGCTCTGCACTCGATGAGGGAGATGCTCCATCAAAG
TGGTTTTCAGATTATCTTGGAAAACCCAGTCGTTTGGTTCGCTTTAATCCAGCTTCACAA
ACAAGGAAAGTAGATCCCAATTATGGTCCTGGACATCAAATCATGTTTTCGGATGAGTTC
CCCTACATGTTGATATCTCAGGGATCTTTGGATGCTTTGAATAAAGTTCTCAAAGAACCC
GTATCAATTAATCGCTTTAGACCTAACATTCTTGTTGATGGCTGCGAACCATTCTCTGAG
GATTTGTGGACTGAGATCGAAATAGACAAGTTCATATTTCAGGGTGTTAGGCTATGTGCC
CGTTGCAAGGAGACTGGAATTGCAGGACCTGAGCTGAATGAAACTCTAAAGAAAATGAGA
TCAGATACTGTCCTGCGTCCAAACCATAAACAAAAGGGAAAAATTTTCTTTGGCCAGAAT
TTGGTTTGGAAGAACATAGCAGCTGAAGGGAAGGGGAAAATCATAAAAGTGGGAGATAAA
GTCAAGGTTCTGGGGAAGGTTGCCTCTGTAGCGGAAGCAGTAGTTTGA
>FGENESH: 1 8 exon (s) 2301 - 4379 295 aa, chain -
MAAVSAIFIYPIKSCRGISVPQAPLTPTGFRWDRQWLVVNSKGRAYTQRVEPKLALVQVD
LPNEAFFDDWSPSKSSFLVIKAPGMDVLKVPLTQPPYNADGVSVWEWSGSALDEGDAPSK
WFSDYLGKPSRLVRFNPASQTRKVDPNYGPGHQIMFSDEFPYMLISQGSLDALNKVLKEP
VSINRFRPNILVDGCEPFSEDLWTEIEIDKFIFQGVRLCARCKETGIAGPELNETLKKMR
SDTVLRPNHKQKGKIFFGQNLVWKNIAAEGKGKIIKVGDKVKVLGKVASVAEAVV
```

Figure 1S. Identification of the *CsARC* gene using the FGENESH program.

A fragment of chomosome 6 of the *Cucumis sativus* cultivar 9930, homologous to the nucleotide sequences of *Arabidopsis AtARC1* (AT1G30910) and *AtARC2* (AT5G44720) [22] acording to BLASTn, was used for the FGENESH

analysis. The predicted *CsARC* coding sequence is located on the negative strand of DNA. It consists of 8 exons with a total length of 888 bp, and the predicted protein sequence is composed of 295 aa.

```
CLUSTAL O(1.2.4) multiple sequence alignment


CrARC1          ---------------------------------------------------ATGCTCACA      9
AtARC2          ------------------------------------------ATGGAGGAAGGTCTAAAG      18
AtARC1          ATGGAGAATACTTTGTCGCCGTTGTTGTCACCGTCGCCGGCAAGTGAAGTCGCTGCGAGA      60
CsARC           ---------------------------------------------------ATGGCGGCT      9


CrARC1          ATCGGCGGTCTTAACATTTATCCCATCAAGTCATGTCGCGGTGTGTCCCTCCAGAGCGCT      69
AtARC2          ATTCAATCTTTGGTCATTTATCCGATCAAATCTTGCCGTGGAATCTCTGTTCCTCAGGCT      78
AtARC1          GTATCGTCTTTGTTCGTTTATCCGATCAAATCATGTCGAGGAATTTCTCTGTCTCAGGCA      120
CsARC           GTGTCTGCAATCTTCATTTACCCAATCAAATCATGTCGCGGTATTTCAGTTCCCCAAGCA      69
                 *        *   * **** ** ***** ** ** ** **  * **  *        **

CrARC1          ATGCTCACGGAATCAGGCCTGGCATACGACCGCGAATGGATGGTGGTGCGTGAGGACACG      129
AtARC2          ACCGTTACTCATACTGGTTTTCAGTGGGACCGGTATTGGTTAGTTGTTAACTATAAAGGA      138
AtARC1          GCTCTCACTCCCACCGGATTTCGATGGGATCGAAACTGGTTGATTGTGAATTCTAAAGGA      180
CsARC           CCTCTTACCCCTACTGGATTTCGATGGGACCGTCAATGGTTGGTTGTCAACTCTAAAGGA      129
                    * **     * **  *    *  ** **  * *** *  * **        *

CrARC1          GGCAAGTTCATCTCGCAGCGAGAGAAGGGGCTGCTGGCACTGGTGCAGGTGTCGCTGCCC      189
AtARC2          AGAGC---ATACACTCAAAGAGTTGAGCCAACCCTTGCTCTTGTTGAATCGGAGTTGCCT      195
AtARC1          AGAGG---ATTGACTCAGAGAGTGGAACCAAAGCTTTCTTTGATTGAAGTAGAAATGCCT      237
CsARC           AGGGC---TTACACTCAGAGAGTCGAGCCTAAGCTTGCTCTCGTCCAGGTCGACCTCCCA      186
                 *           * **  ***   *       **  *  *  *  *        * **

CrARC1          GTGGAGGCGCTGGCGGCGGCGCAGTGGGGCGCCGCCGGCCTGCCGCCGGACGCCGCGCTG      249
AtARC2          AAGGAAGCCTTCTTGGAA---------------GATTGGGAGCCAACAAATGACTCATTG      240
AtARC1          AAGCATGCGTTTGGAGAG---------------GACTGGGAGCCTGAGAAGAGCTCTAAC      282
CsARC           AACGAAGCTTTCTTTGAT---------------GACTGGAGCCCTTCTAAATCCTCTTTT      231
                    * **  *    *                 *   *    **     *   * *

CrARC1          ACGGTGACCGCGCCCGGAATGACGGCGCCGCTCAAGGTGCCGCTGGCGCGCCGCCCCGAC      309
AtARC2          TTGGTGATAAGAGCTCCTGGTATGAGTCCGTTAAAGATACCGTTGACTAGG---CCAAGC      297
AtARC1          ATGGTGGTTAGAGCTCCTGGTATGGATGCCCTTAAGGTCTCCCTAGCTAAA---CCCGAC      339
CsARC           CTCGTCATTAAAGCTCCTGGGATGGATGTCTTAAAGGTTCCCCTTACTCAA---CCTCCG      288
                   **        *       * *       * *** *  *  *  *       **

CrARC1          AGCGAGGCCAAGAAGGTGACGGTATGGGAGTGGACCGGCACCGCCACAGACGAGGGCCCC      369
AtARC2          TCGGTAGCAGAAGGTGTGTCGATGTGGGAATGGTCTGGCTCTGCTTTTGATGAAGGAGAA      357
AtARC1          AAAATAGCAGACGGTGTCTCAGTTTGGGAGTGGTCTGGCTCCGCACTAGATGAAGGAGAA      399
CsARC           TATAATGCTGACGGTGTCTCTGTCTGGGAATGGTCTGGCTCTGCACTCGATGAGGGAGAT      348
                      **  *    **  *  * ***** *** * *** * **    ** ** **

CrARC1          GACGCCGCCGCATGGTTCACCACCTACCTGGGACTGCCCTGTCGCCTGGTGCGCTACGTC      429
AtARC2          GAAGCTGCGAAATGGTTTTCAGATTATCTTGGAAAACAAAGCCGTTTGGTTCGGTTTAAT      417
AtARC1          GAAGCATCTCAGTGGTTTACAAACTTTGTTGGGAAGCCTTGTCGACTTGTTCGTTTTAAT      459
CsARC           GCTCCATCAAAGTGGTTTTCAGATTATCTTGGAAAACCCAGTCGTTTGGTTCGCTTTAAT      408
                *   *  *    *****  *    *   * **     *   * **  * ** ** *

CrARC1          GGCAGCGGCAGTAGCAGCGGCAGTGCAGCCGGTGGCAGTAGCAGCGGCGGCGGGTTGCCG      489
AtARC2          AAAGATACTGAAACTAG-------------------------------------------      434
AtARC1          TCAGCCTATGAGACTAG-------------------------------------------      476
CsARC           CCAGCTTCACAAACAAG-------------------------------------------      425
                            *  **

CrARC1          GTCGTGCGCAACACGGAGCCGGAGTTCGCGGTCAAGTACGAGACGCGCTTCAGCGACGGC      549
AtARC2          --------ACCTTCACCTCCTGAGTTTGCAGCGGGTTACTCTACAACATTCATGGATATG      486
AtARC1          --------GCCTGTGGATCCAAATTATGCTCCAGGTCACATTGCGATGTTCTCGGATATG      528
CsARC           --------GAAAGTAGATCCCAATTATGGTCCTGGACATCAAATCATGTTTTCGGATGAG      477
                                  **  * *  *         *          **    **

CrARC1          TACCCCATGCTGATAGTGACACAGGCGGCGCTGGCGGATCTGAACACCAAGCTGGCGGAG      609
AtARC2          TTTCCGTTTTTGGTTGCATCTCAGGGTTCTTTAGACCATTTGAATACCCTTCTGCCAGAA      546
AtARC1          TATCCATTCTTGCTTATATCACAGGGTTCGCTTGATTCCCTGAATAAGCTTCTCAAGGAG      588
CsARC           TTCCCCTACATGTTGATATCTCAGGGATCTTTGGATGCTTTGAATAAAGTTCTCAAAGAA      537
                *  **     ** *     * ****   *  * *      **** *     **    **

CrARC1          CCGCTGCCCATGAACCGCTTCCGGCCCAACATTGAGGTGGCCGGCGCCTCGCCCTGGGCT      669
AtARC2          CCGGTGCCTATAAACCGTTTTAGACCCAACATTCTTGTTGATAATTGTGATCCTTTCGGT      606
AtARC1          CCTGTACCGATCAACCGATTTAGACCCAACATCTTTGTTGATGGATGTGAACCATTTGCT      648
CsARC           CCCGTATCAATTAATCGCTTTAGACCTAACATTCTTGTTGATGGCTGCGAACCATTCTCT      597
                **  *  * ** ** ** ** **  * ** *****    ** *           ** *    *

CrARC1          GAGGACACCTGGCGCGACATCGATGTGGCCTGCGGCGCCGACGGCCGCACCCTGCGCCTC      729
AtARC2          GAAGATCTTTGGGATGAAATCAAAATAAACGATTTAGT------------------CTTC      648
AtARC1          GAAGACTTATGGACAGAGATCCTTATAAACGGTTTCAC------------------CTTT      690
CsARC           GAGGATTTGTGGACTGAGATCGAAATAGACAAGTTCAT------------------ATTT      639
                ** **    ***   ** ***    *   *                            *

CrARC1          ACCTTCGTCAAGCCCTGCTCCCGCTGCAAGGTGACCACCATCAACCAGGCCACGGGGGAG      789
AtARC2          CAAGGAGTTAGGCTATGTAGCCGCTGCAAGGTACCAACTGTGAATCAAGAAACCGGGGTT      708
AtARC1          CACGGTGTGAAATTATGCTCTCGCTGCAAGGTACCTACGATTAGTCAAGAAACTGGTATT      750
CsARC           CAGGGTGTTAGGCTATGTGCCCGTTGCAAGGAGACTGGAA------------------TT      681
                      ** *     **    ** *******   *

CrARC1          GC---GGGCGATGAGCCGCTGGACACACTGGGAGAGTTCAGGACGGGCAAGGTGCTGGGA      846
AtARC2          ATGGGTAAAGCAGAACCAACTGAAACTCTGATGAAATTCAGATCAGACAATGTCTTAATG      768
AtARC1          GG---AGGTCAAGAGCCAATTGAGACTTTGAGGACTTTCAGGTCAGACAAAGTTTTACAG      807
CsARC           GC---AGGACCTGAGCTGAATGAAACTCTAAAGAAAATGAGATCAGATACTGTCCTGCGT      738
                            ** *     ** **  *        * **  * *  *  **  *

CrARC1          TGGAACGCCAAACAGAAGCCGTGGACGCACGCAGTGTTCTTCGGCTGGAATGTGGTGTCC      906
AtARC2          CCTGACAAGAAACCGCGTG---------GAAAGGTTTCTTTGGCAAGGAGATGGTTTGG      819
AtARC1          CCAAAGAGTAAACCACACG---------GAAAGATATACTTTGGGCAGAACATGGTTTGG      858
CsARC           CCAAACCATAAACAAAAGG---------GAAAAATTTCTTTGGCCAGAATTTGGTTTGG      789
                    *    ****                     * * *** **   * *  **** *

CrARC1          CGCACGCCGG--------------------------GGCTGCTGAGCCTTGGCGACACC      939
AtARC2          AATTGGAACTTAACCAACACTGAAGGCGAAGGAAAGAAAACAATCAAAGTTGGTGATACC      879
AtARC1          AAAGACGGATTCGGCGATGGAATTGG---------AAAAACAATTGAAATTGGTGATTCC      909
CsARC           AAGAACATAGCAGCTGAAGGGAAGGG---------GAAAATCATAAAAGTGGGAGATAAA      840
                                                           *     * ** **

CrARC1          CTCACGCCCGTCACGCAGCAGCAGCCCGCGGACCTGGTGCCAGCGCCGTGA 990
AtARC2          ATCTCAGTCATAAGGAAGATCC---CTTCCAGAGCTGAAGCAGCTGTTTAA 927
AtARC1          GTTGTTGTCCTTCGAAAACTCT---CATCTCCTGCTGAAGCAGCAACTTGA 957
CsARC           GTCAAGGTTCTGGGGAAGGTTG---CCTCTGTAGCGGAAGCAGTAGTTTGA 888
                 *        *     *        *  *       *   ***     * *
```

Figure 2S. Comparison of *AtARC1* (AT1G30910), *AtARC2* (AT5G44720), *CrARC1* (JF940524) from *Chlamydomonas reinhardtii*, and predicted *CsARC* CDS sequences using Clustal Omega (http://clustal.org/omega/). Asterisks indicate positions with a single, fully conserved nucleotide in all aligned sequences.

Percent Identity Matrix - created by Clustal2.1

	CrARC1	AtARC2	AtARC1	CsARC
1: CrARC1	100.00	47.30	47.41	47.24
2: AtARC2	47.30	100.00	65.68	65.20
3: AtARC1	47.41	65.68	100.00	67.45
4: CsARC	47.24	65.20	67.45	100.00

Figure 3S. Estimation of sequence identity of *AtARC1*, *AtARC2*, *CrARC1*, and *CsARC* CDS using Clustal Omega. Values presented are expressed as percentages.

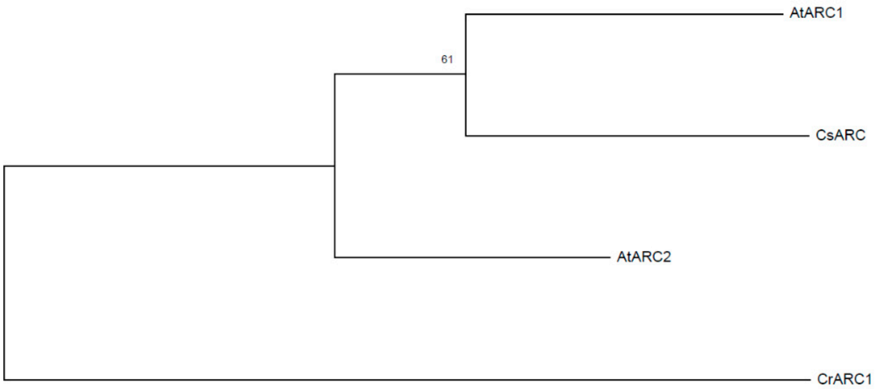

Figure 4S. Phylogenetic tree of AtARC1, AtARC2, CrARC1, and predicted CsARC aa sequences. Multiple alignments were performed using ClustalOmega. The tree was constructed using MEGAX software (https://www.megasoftware.net/) by the maximum likelihood method with 1000 bootstrap replicates.
